# Protocol for cyclic DNA-damaging treatments to generate high mutational burden in the human A549 cell line

**DOI:** 10.1016/j.xpro.2026.104842

**Published:** 2026-09-18

**Authors:** Gergely Imre, Szilvia Juhász

**Affiliations:** 1 HCEMM Cancer Microbiome Core Group, 6728 Szeged, Hungary; 2 Synthetic and Systems Biology Unit, Institute of Biochemistry, HUN-REN Biological Research Centre, Szeged, Hungary

**Keywords:** Cell Biology, Cancer, Genomics, Molecular Biology

## Abstract

Here, we present a protocol that describes iterative exposure of the human lung cancer cell line A549 to DNA-damaging agents to progressively increase mutational burden. We describe steps for controlled cytotoxic dosing, metabolic activation, and repeated treatment cycles of up to 70 iterations, followed by clonal isolation. This approach enables the generation of highly mutated cell populations suitable for downstream genomic analyses.

For complete details on the use and execution of this protocol, please refer to Juhász et al.^1^

## Highlights

• Steps for iterative DNA-damaging treatments up to 70 cycles
• Instructions for maintaining cytotoxicity and recovery across cycles
• Guidance for clonal expansion and isolation of treated cells
• Steps for preparing samples for whole-genome sequencing

## Abstract (editor highlights)

Publisher’s note: Undertaking any experimental protocol requires adherence to local institutional guidelines for laboratory safety and ethics.

## Graphical abstract

**Figure:**
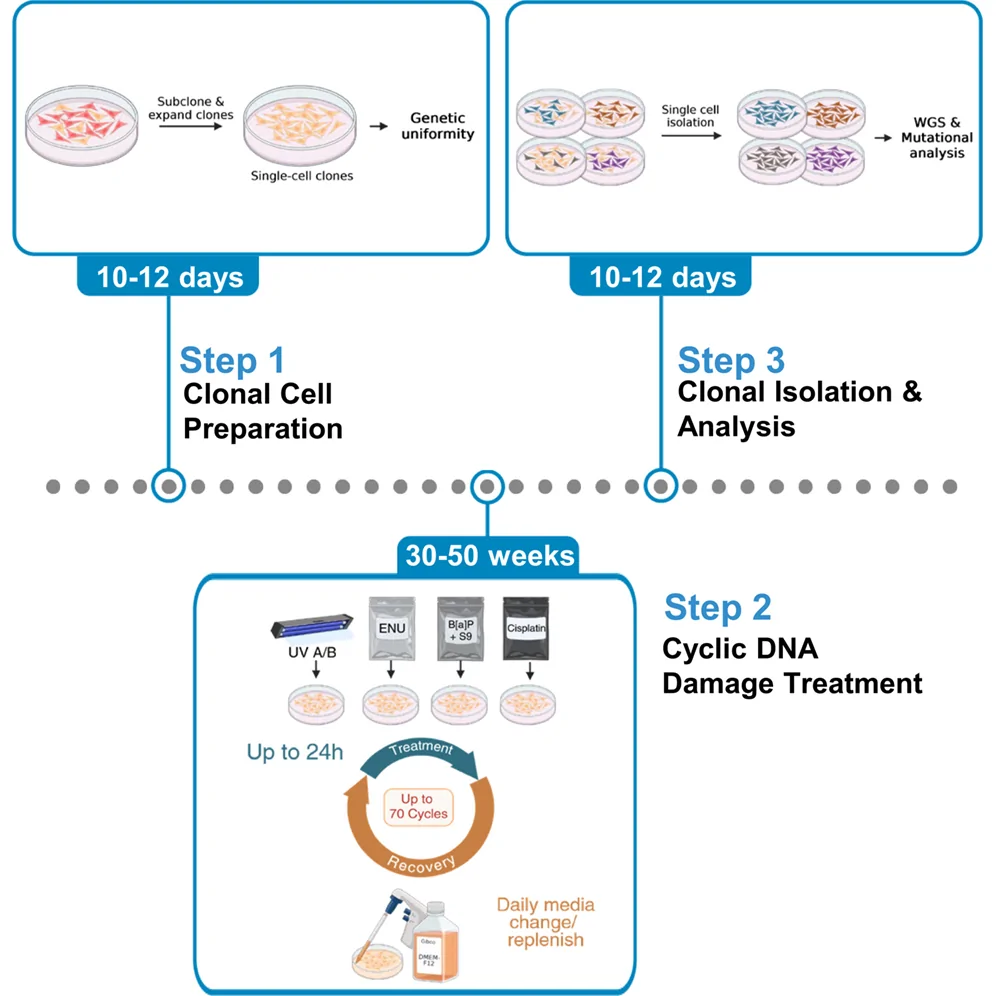

## Before you begin

**Figure 1:**
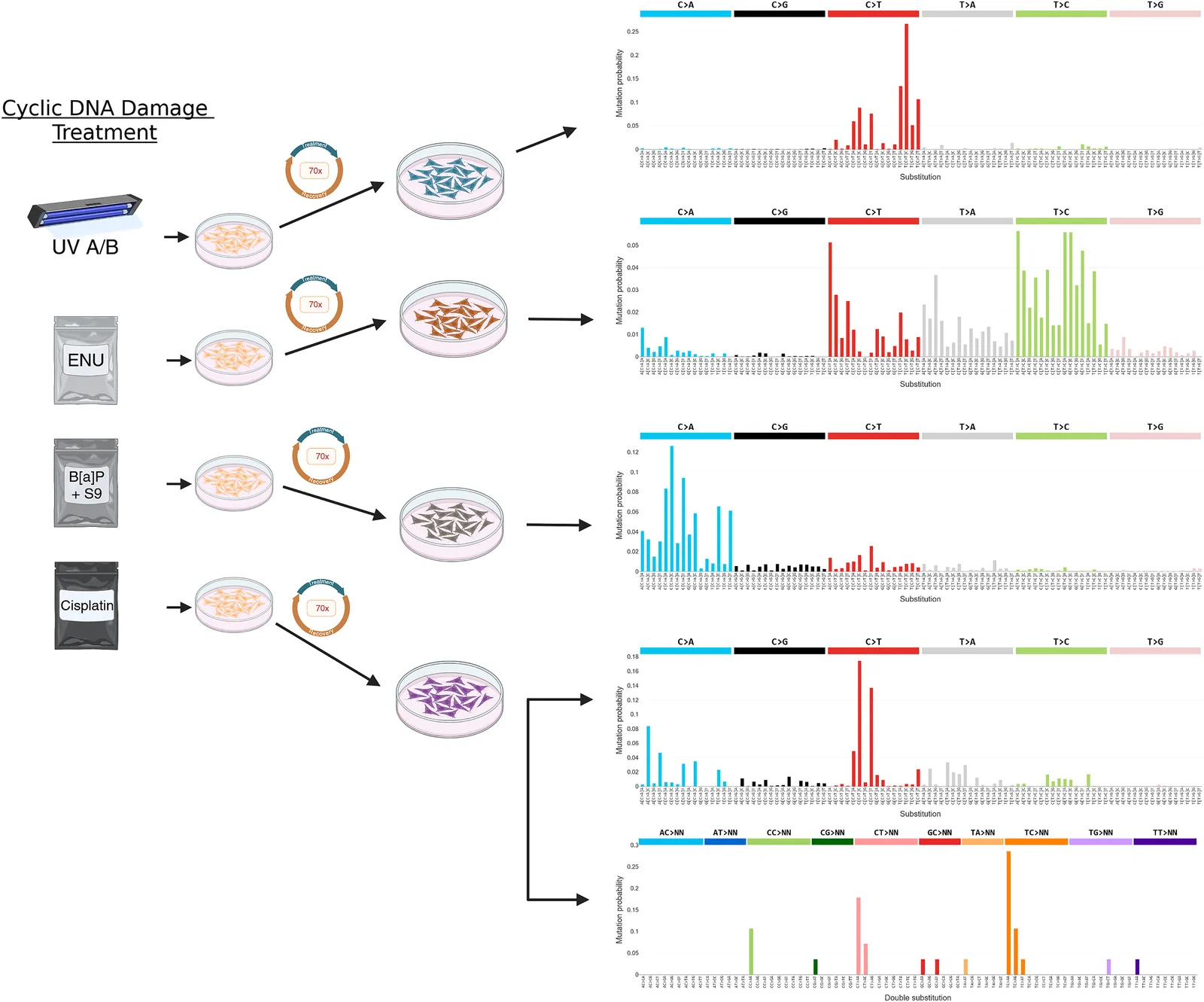
Iterative DNA damaging treatments drive mutation accumulation and distinct mutational signature profiles Created with BioRender.com.

This protocol describes cyclic treatment of the human lung adenocarcinoma cell line A549 using DNA damaging agents to induce progressive accumulation of mutations (Figure 1).^1^ The workflow builds upon the mutagen exposure strategy developed by Kucab et al.^2^ and extends it through long-term iterative treatment cycles to substantially increase mutational burden. Although the protocol was optimized and experimentally validated in A549 cells, the general approach is expected to be applicable to other mammalian cell lines following optimization of treatment conditions, particularly the concentration of each DNA damaging agent required to achieve approximately 40%–60% cytotoxicity.

1. Culture A549 cell line in DMEM:F12 supplemented with 10% FBS and antibiotics at 37°C and 5% CO_2_.
2. Ensure cells are mycoplasma-free and in exponential growth phase prior to treatment.
3. Determine dose-response curves for each DNA damaging agent in the selected cell line and identify the concentration that produces approximately 40%–60% cytotoxicity.
  ***Note:*** As sensitivity to DNA damaging agents varies among cell lines, concentrations established for A549 cells should not be directly transferred without prior optimization. The dose optimization strategy described by Kucab et al.^2^ provides a useful framework for adapting this protocol to additional cellular models.
  a. Seed cells under the same conditions that will be used during the iterative treatment protocol.
  b. Prepare a serial dilution of each DNA damaging agent covering at least one order of magnitude (typically 3–6 concentrations).
  c. Treat cells using the same exposure time described in the protocol.
  d. Following treatment, allow cells to recover under standard culture conditions and determine cell viability using an appropriate viability assay (e.g., CellTiter-Glo, MTT, resazurin, crystal violet staining, or direct cell counting).
  e. Select the concentration that consistently results in approximately 40%–60% cytotoxicity (equivalent to 60%–40% cell survival).
    **CRITICAL:** Accurate cytotoxicity calibration is essential to maintain long-term survival across multiple treatment cycles.
4. Prepare all reagents fresh before treatment cycles.

### Innovation

This protocol establishes a long-term, iterative DNA damaging treatment strategy (up to 70 cycles) that enables controlled and progressive accumulation of mutations in cultured human cells. In contrast to conventional single-exposure or short-term mutagenesis approaches, this method combines defined cytotoxic stress with recovery phases, allowing sustained cell survival while modeling genome instability as a time-dependent, evolutionary process.

A key advantage of this approach is its ability to generate agent dependent mutation burdens while preserving treatment-associated mutational signature specificity. After 70 iterative treatment cycles in this study, the analyzed clones contained approximately 1,000 to nearly 480,000 filtered acquired single nucleotide variants (SNVs) per clonal genome. This represents a substantial increase in mutation burden compared with conventional single exposure or short-term mutagenesis approaches and provides sufficient mutation counts for efficient identification of mutational processes. This protocol is based on whole-genome sequencing (WGS), mutation accumulation is reported as the total number of acquired somatic SNVs per clone rather than as tumour mutational burden (mutations/Mb), which is primarily used for clinical tumour profiling.

The protocol incorporates a liver S9 metabolic activation system, a widely used in vitro enzyme preparation containing phase I metabolic enzymes (primarily cytochrome P450 enzymes) that mimics hepatic xenobiotic metabolism. The S9 system enables metabolic activation of pro-mutagens, such as benz[a]pyrene, into DNA reactive intermediates, thereby broadening the spectrum of DNA damaging agents that can be investigated.^2^ Together, these features provide a reproducible framework for generating highly mutated cell populations suitable for high-resolution analysis of mutational signatures, including amino acid substitution patterns, and for studying genome instability in a controlled experimental setting.

## Key resources table

**Table:** 

REAGENT or RESOURCE	SOURCE	IDENTIFIER
**Chemicals, peptides, and recombinant proteins**		

N-ethyl-N-nitrosourea (ENU)	Sigma-Aldrich	N3385-1G
Cisplatin	Sigma-Aldrich	P4394-100MG
Benz[a]pyrene	Sigma-Aldrich	B1760-250MG
S9 fraction (Aroclor-1254 induced hamster liver)	Moltox	#15-03SL.5
DL-isocitric acid trisodium salt hydrate	Sigma-Aldrich	I2002
NADP	Roche	10128031001

**Critical commercial assays**		

Genomic DNA extraction kit	Lonza	N/A

**Deposited data**		

Whole-genome sequencing data for the publicly deposited subset	Juhász et al.^1^	ENA: PRJEB102539
GRCh38 reference genome, UCSC hg38 analysis set	UCSC Genome Browser^3^	hg38.analysisSet.fa; GenBank: GCA_000001405.15; https://hgdownload.soe.ucsc.edu/goldenPath/hg38/bigZips/analysisSet/
ENCODE blacklist v2 (hg38)	Amemiya et al.^4^	https://github.com/Boyle-Lab/Blacklist
SIGNAL experimental exposure signatures (n = 54; MutationalPatterns v3.22.0)	Degasperi et al.^5^	https://signal.mutationalsignatures.com
COSMIC SBS signatures (n = 60; MutationalPatterns v3.22.0)	Alexandrov et al.^6^	https://cancer.sanger.ac.uk/signatures/

**Experimental models: cell lines**		

A549 HLA subtype panel cell lines	Sigma-Aldrich	HLA003-1KT

**Software and algorithms**		

BWA v0.7.17	Li and Durbin^7^	https://github.com/lh3/bwa
Sambamba v0.7.1	Tarasov et al.^8^	https://lomereiter.github.io/sambamba
Picard v2.18.9	Broad Institute^9^	https://broadinstitute.github.io/picard
SAMtools v1.13	Danecek et al.^10^	https://www.htslib.org
IsoMut	Pipek et al.^11^	https://github.com/riblidezso/isomut
Python 2.7	Python Software Foundation	https://www.python.org
R v4.6.1	R Core Team^12^	https://www.r-project.org
Bioconductor v3.23	Huber et al.^13^	https://www.bioconductor.org
GenomicRanges v1.64.0	Lawrence et al.^14^	Bioconductor
rtracklayer v1.72.0	Lawrence et al.^15^	Bioconductor
Biostrings v2.80.1	Pagès et al.^16^	Bioconductor
GenomeInfoDb v1.48.0	Arora et al.^17^	Bioconductor
MutationalPatterns v3.22.0	Manders et al.^18^	Bioconductor
BSgenome.Hsapiens.UCSC.hg38 v1.4.5	Pagès^19^^,^^20^	Bioconductor
ggplot2 v4.0.3	Wickham^21^	CRAN
ggdendro v0.2.0	de Vries and Ripley^22^	https://cran.r-project.org/package=ggdendro
RColorBrewer v1.1-3	Neuwirth^23^	https://cran.r-project.org/package=RColorBrewer
Joint IsoMut SNV call set and reported analysis outputs	This paper	Zenodo: https://doi.org/10.5281/zenodo.22001044
Analysis scripts for this protocol (v1.0.0)	This paper	GitHub: https://github.com/imgergely/WGS-signatures; Zenodo: https://doi.org/10.5281/zenodo.22001044
Ubuntu Linux 22.04.5 LTS, x86_64	Canonical	https://ubuntu.com/

**Other**		

SSRsimulated solar radiation (SSR) machine	Vilber Lourmat VL-6.LM	N/A
Qubit	Thermo Fisher Scientific	N/A

## Materials and equipment

**Table:** S9 mix preparation

Reagent	Final concentration	Amount
Aroclor-1254-induced male Golden Syrian hamster liver	0.25 V/V %	25 μL
NADP	3 mM	300 μL of 100 mM stock
DL-Isocitric acid trisodium salt hydrate	15 mM	1.5 mL of 100 mM stock
Growth/culture medium	N/A	8.175 mL
**Total**	**N/A**	**10 mL**

***Note:*** Prepare this solution fresh.

## Step-by-step method details

### Preparation of clonal starting populations

**Timing: 10–12 days**

This section describes the isolation and expansion of single-cell-derived A549 clones used as genetically defined starting populations for iterative DNA damage treatment. Establishing a monoclonal starting population minimizes pre-existing genetic heterogeneity and provides a consistent baseline for subsequent mutation accumulation.

1. Detach cells using trypsin and generate a single-cell suspension.
2. Determine the cell concentration using a hemocytometer or automated cell counter and dilute the suspension to 5–10 cells/mL in complete growth medium.
  a. Seed 10 mL of the diluted suspension into each 10-cm tissue culture dish, resulting in approximately 50–100 cells per dish.
3. Replace medium daily and allow colonies to grow for 10–12 days.
4. Examine the dishes daily using an inverted phase-contrast microscope.
  a. After approximately 10–12 days, identify well-separated colonies that are clearly derived from a single founder cell and do not overlap with neighboring colonies.
  b. Mark the selected colonies on the underside of the dish.
  c. Using a sterile 10 μL pipette tip, gently scrape and aspirate the cells from a single, well-isolated colony, taking care not to disturb adjacent colonies.
  d. Transfer the collected cells into one well of a 24-well plate containing complete growth medium.
5. Expand selected clones sequentially into 24-well, 6-well, and larger culture formats.
6. Prepare cryopreserved stocks of each clone to serve as baseline controls for downstream sequencing.

**CRITICAL:** Starting from monoclonal populations is essential to ensure that observed mutations arise from treatment rather than pre-existing heterogeneity.

***Note:*** If cells tend to grow in clusters or remain aggregated after routine trypsinization, gently pipette the suspension up and down several times to disrupt cell clumps. If necessary, extend the trypsinization time by 1–2 min, while avoiding over-trypsinization that may compromise cell viability. Accurate single-cell suspensions are essential for obtaining colonies derived from individual cells. In addition, avoid selecting colonies that are too close to each other, as mixed populations may compromise downstream analysis.

### Preparation of treatment media

**Timing: 30–60 min**

This section describes the preparation of fresh treatment media containing the selected DNA damaging agents at the concentrations used for iterative treatment. Preparing treatment media immediately before exposure helps maintain consistent dosing across treatment cycles.

7. Prepare fresh treatment media immediately before each exposure using the conditions specified in the preparation of treatment media section:
  a. N-Ethyl-N-nitrosourea (ENU): Prepare a stock solution in DMSO and dilute to a final concentration of 400 μM in complete culture medium immediately before treatment.
  b. Cisplatin: Prepare a stock solution in sterile 0.9% NaCl and dilute to a final concentration of 5 μM in complete culture medium immediately before use.
  c. Benz[a]pyrene (BaP): Prepare a stock solution in DMSO and dilute to a final concentration of 0.39 μM in complete culture medium containing the S9 metabolic activation mix.
8. For compounds requiring metabolic activation, prepare the S9 mix and supplement the treatment medium. In this study, BaP was added to the S9-containing medium at a final concentration of 0.39 μM.

**CRITICAL:** DNA damaging agents are unstable; always prepare fresh solutions and avoid prolonged storage.

***Note:*** The concentrations described in this protocol were optimized for A549 cells. When applying this protocol to other cell lines, the optimal concentration of each DNA damaging agent should be determined by dose-response experiments to identify conditions producing approximately 40%–60% cytotoxicity. A comprehensive panel of optimized mutagen concentrations for human cell line is provided by Kucab et al.,^2^ which can serve as a useful starting point for experimental optimization. To ensure that the selected concentration accurately reflects the experimental conditions used during iterative treatment, dose-response experiments should preferably be performed using the same culture format (6-well plates), seeding density, medium volume, and exposure conditions as the main protocol.

### Execution of a single treatment cycle

**Timing:up to 24 h exposure, followed by 2–4 days of recovery**

This section describes the application of DNA damaging agents to cultured cells under controlled exposure conditions, followed by immediate medium replacement or recovery. Consistent treatment conditions are maintained across cycles to support progressive mutation accumulation while preserving sufficient cell viability for subsequent treatments.

9. Seed cells into 6-well plates at 2.0–2.5 × 10^5^ A549 cells per well density (one plate per treatment condition) to reach ∼70–80% confluency at treatment.
10. Replace culture medium with treatment medium.
11. Treat BaP samples under S9-dependent conditions.
  a. Incubate cells with S9-containing medium for 3 h.
  b. After BaP+S9 treatment, replace the treatment medium with pre-warmed complete growth medium and incubate the cells under standard culture conditions (37°C, 5% CO_2_) for a 2-4-day recovery period before initiating the next treatment.
12. Treat cells with non-S9 compounds (cisplatin or ENU).
  a. Incubate cells for up to 24 h with the treatment medium.
  b. After cisplatin or ENU treatment, replace the treatment medium with pre-warmed complete growth medium and incubate the cells under standard culture conditions (37°C, 5% CO_2_) for a 2-4-day recovery period before initiating the next treatment.
13. Apply simulated solar radiation (SSR).
  a. Before irradiation, remove the culture medium from the cells.
    ***Note:*** The SSR output consisted of 10% UVB (295–315 nm) and 90% UVA (315–400 nm), with a total dose of 1.25 J.
    ***Note:*** At the irradiance used in this study, the required dose was delivered as a short pulse lasting only a few seconds.
  b. Immediately after UV irradiation, add pre-warmed complete growth medium.
  c. Incubate the cells under standard culture conditions (37°C, 5% CO_2_) for a 2- 4-day recovery period before initiating the next treatment cycle.
    **CRITICAL:** Ensure uniform treatment conditions across all samples (e.g., equal media volume, consistent irradiation distance).
    ***Note:*** Variability in exposure is a major source of experimental inconsistency.
    **Solution (if needed):** If excessive cell death occurs, reduce exposure time or concentration while maintaining ∼40%–60% cytotoxicity.

### Recovery phase

**Timing: 2–4 days**

Following treatment, cells are allowed to recover and proliferate before the next cycle.

14. Replace treatment medium with fresh growth medium.
15. Maintain cells under standard conditions until they reach ∼70%–80% confluency.
16. Replace medium daily to remove residual compounds.

**CRITICAL:** Do not initiate the next cycle until cells exhibit stable proliferation.

***Note:*** Insufficient recovery reduces long-term survival and may lead to population collapse.

**Solution (if needed):** Extend recovery time if growth is delayed or cell density remains low.

### Iterative treatment cycles (up to 70 cycles)

**Timing: 3–5 days per cycle; approximately 30–50 weeks total**

Repeated cycles progressively increase mutation accumulation while maintaining viable cell populations through alternating treatment and recovery phases.

17. Repeat steps 9–16 for up to 70 cycles.
18. Maintain consistent dosing corresponding to 40%–60% cytotoxicity across all cycles.
19. Passage cells as needed to avoid over-confluency.
20. Cryopreserve aliquots at regular intervals (e.g., every 5–10 cycles).

**CRITICAL:** Consistent experimental conditions across all treatment cycles are essential for reproducibility. Standardize cell density, treatment timing, exposure conditions, recovery intervals, and media volumes throughout the experiment.

***Note:*** The number of treatment cycles should be selected according to the desired mutation burden and downstream application. In this study, the protocol was experimentally validated for up to 70 iterative treatment cycles, which reproducibly generated approximately 1,000–480,000 filtered acquired SNVs per clonal genome, providing sufficient mutation counts for reliable mutational signature analysis. The minimum number of cycles is not fixed and depends on the experimental objective, the DNA damaging agent, the cell type, and the required mutation burden. Fewer cycles may be sufficient for studies of early mutagenesis, whereas additional cycles increase mutation accumulation but may also enhance clonal selection and alter cellular fitness. Since the relationship between cycle number and mutation burden has not been systematically characterized, no saturation point has been established.

**Solution (if needed)**: If cell viability progressively declines during iterative treatment, extend the recovery period until cells resume normal proliferation or reduce the treatment intensity by lowering the concentration or shortening the exposure time while maintaining the target cytotoxicity (40%–60%).

### Clonal isolation after completion of iterative treatment

**Timing: Approximately 2 weeks**

Following completion of the planned iterative treatment regimen (up to 70 treatment cycles in this study), surviving cells are subjected to single-cell cloning to establish monoclonal populations for downstream genomic analyses, including WGS.

21. Generate single-cell suspensions and seed at low density in 10 cm dishes (50–100 cells per dish).
22. Allow colonies to grow for 10–12 days.
23. Select well-isolated colonies and expand them.
24. Collect cell pellets for genomic DNA extraction for WGS analysis.

**Pause point:** Cells can be cryopreserved before DNA extraction.

**CRITICAL:** Ensure clonal origin of each colony; mixed populations will compromise sequencing results.

***Note:*** Do not perform clonal isolation immediately after the final DNA damaging treatment. Instead, allow cells to complete one recovery phase (typically 2–4 days) under standard culture conditions before initiating single-cell cloning. This recovery period is distinct from the subsequent 10–12-day colony expansion period (Step 22), during which single cells proliferate to form colonies suitable for isolation. In this study, clonal isolation was performed after completion of all planned treatment cycles (70 cycles). If fewer treatment cycles are used to address a specific biological question, clonal isolation should similarly be performed after the final treatment cycle and recovery phase.

### Extraction of the genomic DNA

**Timing: 1 day**

This section describes preparation of high-quality genomic DNA from treated monoclonal cell populations for WGS. Library preparation and sequencing were performed by a commercial sequencing provider.

25. Isolate genomic DNA from expanded monoclonal cell populations using a genomic DNA purification kit according to the manufacturer's instructions (Lonza).
26. Measure DNA concentration using a fluorometric DNA quantification assay (e.g., Qubit) or an equivalent method.
27. Prepare genomic DNA according to the submission requirements of the sequencing provider.

**CRITICAL:** High-quality, high-molecular-weight genomic DNA is essential for accurate variant detection and downstream mutational signature analysis. DNA degradation or contamination may adversely affect sequencing quality and mutation calling.

***Note:*** Prepare purified genomic DNA according to the sequencing provider's submission requirements and submit the samples to Novogene for library preparation and whole-genome sequencing. In this study, libraries were prepared by Novogene and sequenced on an Illumina NovaSeq 6000 using 150 bp paired-end reads, targeting a mean whole-genome sequencing depth of approximately 30× per clone. The sequencing provider performed library preparation and primary sequencing quality control. The resulting BAM files were subsequently used for the downstream computational analysis described below. In addition, researchers performing sequencing in-house should follow the library preparation protocol recommended for their sequencing platform.

### Mutational signature analysis

**Timing: 2–3 days**

This section describes the computational analysis of WGS data from the treated clones, including alignment quality control, identification of treatment-associated single nucleotide variants (SNVs), construction of the 96-channel single-base substitution (SBS96) mutation profiles, and comparison with experimentally derived reference signatures.

***Note:*** Functional annotation of variants and the amino acid substitution analysis derived from these data are outside the scope of this protocol and are described in Juhász et al.^1^ The SAMtools quality control, IsoMut mutation calling, and downstream R analyses were performed on a 64-bit x86_64 system running Ubuntu 22.04.5 LTS. Complete executable implementations are provided as run_samtools_qc.sh, isomut_config.py, and wgs_signature_pipeline.R. The code blocks below are limited to short standalone commands required for file preparation, execution, and validation. Steps 32–39 describe sequential analytical stages implemented in the supplied R script; they are not executed as separate commands.

***Note:*** For users establishing a comparable computational environment, a workstation equipped with a 16-core processor, 64 GB RAM, an NVIDIA GPU with 12 GB VRAM, and at least 2–10 TB of SSD storage is recommended. These specifications provide sufficient computational capacity for the whole-genome sequencing data processing, variant analysis, and downstream mutational signature analyses described in this protocol. GPU acceleration is not essential for the core analytical workflow but may be useful for other computational applications.

***Note:*** The analytical approach is not specific to the agents used here, however, the supplied scripts are configured for the study cohort and must be adapted when analyzing a different sample panel or treatment design. It requires only a panel of clones derived from a single parental population, at least one untreated clone sequenced in the same batch, and short read WGS at comparable depth across all clones.

***Note:*** Two entry points are supported for the computational workflow. To perform an end-to-end analysis of newly generated BAM files, complete steps 28–39. To reproduce the downstream analyses reported in this protocol, use the joint IsoMut SNV call set supplied in the project repository (input/all_SNVs.isomut.gz) and begin at step 32. The supplied scripts are configured for the 42 clone study panel. Analysis of a different sample panel requires corresponding updates to Table_S1_clone_key.csv, the IsoMut exclusion settings, the expected group composition, and the prespecified reference signatures.

28. Align reads to the UCSC hg38 analysis set, coordinate sort the alignments, and mark duplicates.
  a. Align reads with BWA-MEM to the UCSC hg38 analysis set (hg38.analysisSet.fa).
  b. Coordinate sort the alignments with Sambamba.
  c. Mark duplicate reads with Picard.
  d. Require ≥80% of bases at Q30.

***Note:*** In this study, steps 28a–28d were performed by Novogene using a validated standard workflow. Any equivalent workflow producing coordinate sorted, duplicate marked BAM files aligned to the UCSC hg38 analysis set is acceptable. Index the final BAM files during IsoMut input preparation in step 30.

**CRITICAL:** Use the UCSC hg38 analysis set and one chromosome naming convention throughout. The BAM alignments, FASTA supplied to IsoMut, ENCODE blacklist, and BSgenome object used in R must have compatible hg38 coordinates and contig names. In this study, IsoMut used hg38.analysisSet.fa with chr-prefixed names. A naming mismatch can silently remove variants during downstream filtering.

***Note:*** Check the BAM header before proceeding:

samtools view -H bams/sample.final.bam | grep 'ˆ@SQ' | head

# expect SN:chr1 ...

29. Record per-clone mapping and coverage statistics and inspect them before mutation calling. Run the supplied script from the repository root:

chmod +x run_samtools_qc.sh

./run_samtools_qc.sh

***Note:*** The script runs samtools flagstat and samtools coverage for every ∗.final.bam file in bams/. It writes the per-clone outputs to qc/<sample>.flagstat.txt and qc/<sample>.coverage.txt and writes the weighted mean depth summary to qc/mean_depth_summary.tsv.

***Note:*** Use the per-clone samtools flagstat output to obtain total reads, mapping rate, and duplicate rate. Calculate one mean-depth value per clone by averaging the chromosome level meandepth values returned by samtools coverage for chr1-chr22, chrX, and chrY, weighting each value by the corresponding chromosome length: sum(meandepth_i × span_i) / sum(span_i), where span_i = endpos_i - startpos_i + 1. Representative values are given under Expected outcomes; the complete per-clone table is Table S2.

**CRITICAL:** Library size alone does not indicate a usable sample. In this study, one library, BAP2KO3, contained approximately 8.9 × 10^8^ total reads - more than any other library in its treatment group - yet only 7.65% of reads mapped, yielding a mean depth of 0.30×. Mapping rate and mean depth must therefore be inspected per clone before mutation calling. Because IsoMut evaluates genomic positions jointly across the panel, a failed library must be omitted from the IsoMut input panel in step 30. In this study, BAP2KO3 was omitted before IsoMut and was not included in the joint mutation call.

**Pause point:**. BAM files and quality control statistics can be archived at this stage; downstream steps can be resumed at any time.

### Treatment-associated mutation calling with IsoMut

**Timing: Several hours to 1 day, depending on computing resources**

Because all clones originate from the same parental A549 population, inherited variants and pre-existing cell line variants are generally shared across the panel.

***Note:*** IsoMut is designed to detect variants that are frequent in one clone while the other clones retain the reference allele; variants shared by multiple clones are suppressed as background. No matched normal sample is required.

30. Assemble the 42 clone IsoMut input panel.
  a. Include all untreated and treated libraries that passed the step 29 quality control inspection.
  b. In Table S1, set the Protocol_name of every omitted library to omitted, and list its original clone ID in EXCLUDED_SAMPLE_IDS near the top of isomut_config.py.
    ***Note:*** In this study, the excluded library was specified as follows:
    EXCLUDED_SAMPLE_IDS = set(["BAP2KO3"])
    ***Note:*** Excluded BAMs may remain in bams/ so that their quality control files are retained. isomut_config.py uses Table S1 as the cohort definition, passes only the 42 non-omitted BAMs to IsoMut, and stops if Table S1, EXCLUDED_SAMPLE_IDS, or the BAM directory is inconsistent.
  c. Index the reference FASTA and final BAM files before running IsoMut:
    if [[ ! -f reference/hg38.analysisSet.fa.fai ]]; then samtools faidx reference/hg38.analysisSet.fa
    fi
    for bam in bams/∗.final.bam; do if [[ ! -f "${bam}.bai" && ! -f "${bam%.bam}.bai" ]]; then samtools index "$bam" fi done
    ***Note:*** IsoMut requires hg38.analysisSet.fa.fai and BAM indexes for the 42 files passed to the joint call. BAM indexes are not required for the SAMtools quality control commands in step 29. The shell loop indexes every ∗.final.bam file present in bams/, including an excluded library if it remains there for quality control retention. The excluded BAM is not included in params["bam_filenames"].
31. Run IsoMut across the complete analyzed panel in one execution.
  a. Run the supplied configuration with Python 2.7 from the IsoMut package directory after editing the path constants if required:
    python2 isomut_config.py
    **CRITICAL:** All 42 analyzed clones must be called in a single execution. IsoMut identifies clone-specific variants by comparing each clone with all other clones in the panel. Running treatment groups separately changes the comparison panel and therefore changes which variants are retained as clone-specific.
    ***Note:*** The complete executable configuration is supplied as isomut_config.py. Before starting IsoMut, it validates the reference FASTA and index, Table S1 cohort definition, exclusion list, BAM membership, BAM indexes, IsoMut wrapper, and compiled IsoMut executable. It then passes the 42 analyzed BAMs to IsoMut in one joint execution.
    ***Note:*** IsoMut writes separate SNV and indel output tables. This protocol uses all_SNVs.isomut only; indels are not analyzed. The configuration is adapted from the distributed IsoMut example and is run using Python 2.7.
  b. After IsoMut completes, confirm that the output uses the same chr-prefixed naming convention:
    cut -f2 isomut_output/all_SNVs.isomut | sort -u | head
    # expect chr1 ...

### Downstream R analysis

**Timing: <1 h**

This section describes the final downstream analysis of the processed mutation data in R, including data handling, visualization, and generation of summary outputs for mutational signature analysis.

***Note:*** Steps 32–39 describe the sequential analytical stages implemented in the supplied wgs_signature_pipeline.R script. Run the complete downstream analysis once from the repository root:

Rscript wgs_signature_pipeline.R

***Note:*** The script executes all stages in a single R session. The numbered steps below correspond to labeled sections of the script and describe their inputs, analytical operations, validation criteria, and outputs; they are not executed as separate commands.

32. Load, map, and validate the IsoMut SNV call set.

***Note:*** This stage is implemented in the section labeled STEP 32 in wgs_signature_pipeline.R.

***Note:*** The script reads the joint IsoMut SNV table and Table_S1_clone_key.csv, uses Table S1 to map original clone identifiers to protocol display names, excludes the row marked as omitted, and validates the expected clone cohort. It then verifies chr-prefixed hg38 coordinates and canonical single base SNVs before constructing one GRanges object per clone for chr1-chr22, chrX, and chrY.

***Note:*** Raw identifiers use NEU for N-ethyl-N-nitrosourea, whereas the manuscript and figures use ENU. Table S1 provides the mapping from original clone IDs to protocol display names and records the ENA BioSample accession and availability status for each clone.

33. Remove calls overlapping the ENCODE hg38 blacklist v2 and assemble the per-clone mutation count table.

***Note:*** This stage is implemented in the section labeled STEP 33 in wgs_signature_pipeline.R.

***Note:*** The script removes SNVs overlapping the ENCODE hg38 blacklist v2 and records, for each clone, the number of SNVs before and after filtering and the percentage removed. The resulting table is written to results/Table_S3_mutation_counts.csv.

***Note:*** The fraction of calls removed by blacklist filtering is burden dependent; a fixed number of recurrent artifact positions represents a larger portion of a smaller call set.

34. Confirm that the exact 42 clone panel entered the post-IsoMut analysis.

***Note:*** This validation is implemented in the section labeled STEP 34 in wgs_signature_pipeline.R.

***Note:*** The script requires exactly 42 analyzed clones distributed as 9 NT, 9 UV, 7 CP, 8 BAP, and 9 ENU clones. It stops if an omitted, missing, unexpected, or duplicated identifier is detected. After these checks complete, the script writes results/Table_S3_mutation_counts.csv.

***Note:*** BAP2KO3 failed sequencing quality control (7.65% mapped; 0.30× mean depth) and was omitted from the IsoMut input panel before joint mutation calling. All analysed clones mapped at 99.71%–99.88% with 20.31×-36.50× mean depth. No clone was excluded on the basis of mutation burden.

**CRITICAL:** Untreated control clones in this study carry approximately 1 × 10^3^ to 6 × 10^3^ mutations and provide the background against which treatment groups are interpreted. A burden floor would discard valid controls; no minimum burden threshold was applied.

35. Construct and normalize the per-clone SBS96 matrix.

***Note:*** This stage is implemented in the section labeled STEP 35 in wgs_signature_pipeline.R.

***Note:*** The script uses MutationalPatterns::mut_matrix() with BSgenome.Hsapiens.UCSC.hg38 to classify the filtered SNVs into 96 trinucleotide channels. It verifies that the matrix contains 96 rows and normalizes each clone independently by its total filtered SNV count.

36. Derive treatment level SBS96 spectra.

***Note:*** This stage is implemented in the section labeled STEP 36 in wgs_signature_pipeline.R.

***Note:*** For each treatment group, the script calculates the mean normalized per-clone SBS96 profile.

**CRITICAL:** Average the normalized per-clone profiles rather than pooling raw mutation counts. Mutation burden differs substantially among clones, and pooling would weigh high burden clones more strongly in the treatment level spectrum.

37. Load and validate the SIGNAL and COSMIC reference signature matrices.

***Note:*** This stage is implemented in the section labeled STEP 37 in wgs_signature_pipeline.R.

***Note:*** Using MutationalPatterns::get_known_signatures(), the script obtains the 54 SIGNAL experimental exposure signatures and the 60 COSMIC SBS signatures for GRCh38. It verifies that both matrices contain 96 named channels in the same order as the study SBS96 matrix.

***Note:*** Calculations use the native catalogue column names. Validated display labels are applied only when generating the figures.

38. Calculate clone-level cosine similarities to the prespecified SIGNAL references.

**Figure 2:**
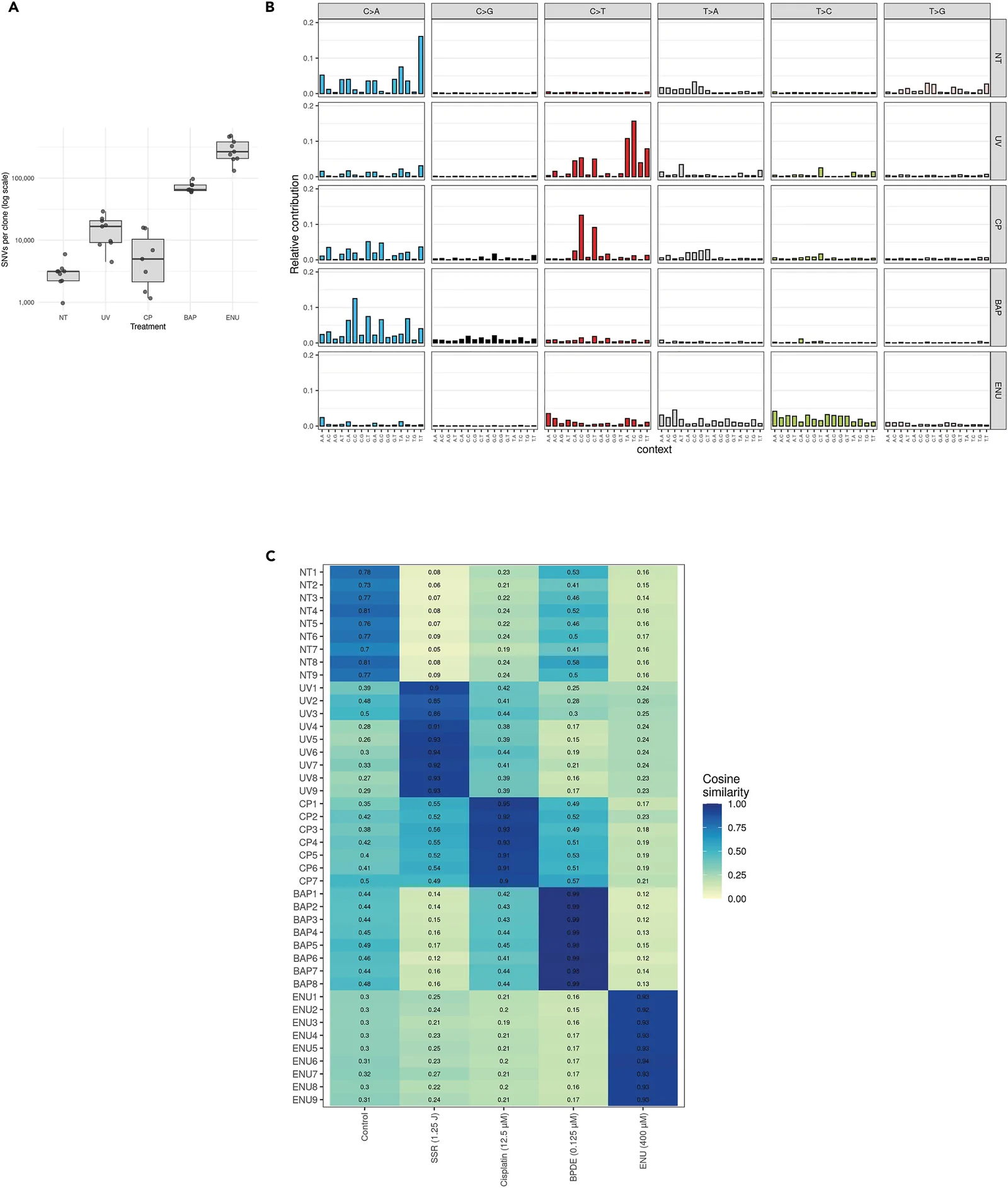
Iterative DNA-damaging treatment generates agent dependent mutational burdens that recapitulate known treatment-associated mutational signatures (A) Number of filtered somatic SNVs per clone after 70 treatment cycles, grouped by treatment (n = 9 NT, 9 UV, 7 CP, 8 BAP, 9 ENU). Each point is one independently expanded clone; boxes indicate the median and interquartile range. Boxplot statistics are calculated on log10-transformed SNV counts. The central line denotes the median, the box spans the interquartile range, and whiskers extend to the most extreme observations within 1.5 times the interquartile range of the lower and upper hinges on this scale. All clones are shown as individual points. (B) Mean SBS96 profile for each treatment, calculated as the mean of per-clone proportions. (C) Cosine similarity between each clone’s SBS96 profile and the five prespecified experimental reference signatures from the SIGNAL database. NT, untreated control; UV, simulated solar radiation; CP, cisplatin; BAP, benzo[a]pyrene + S9; ENU, N-ethyl-N-nitrosourea.

***Note:*** This stage is implemented in the section labeled STEP 38 in wgs_signature_pipeline.R.

***Note:*** The reference mapping is defined in the script before the similarity results are examined. Each reference must resolve to exactly one native SIGNAL catalogue column; the script stops if a reference is absent or resolves ambiguously.

***Note:*** Cosine similarity is calculated between every clone and the complete SIGNAL exposure catalogue. Figure 2C displays the five prespecified references: Control, SSR (1.25 J), Cisplatin (12.5 μM), BPDE (0.125 μM), and ENU (400 μM).

***Note:*** Cosine similarity is descriptive and has no universal attribution threshold. Interpret the prespecified experimental reference in the context of the other signatures in the same catalogue.

39. Generate and inspect the mutation count and mutational signature outputs.
  ***Note:*** This stage is implemented in the section labeled STEP 39 in wgs_signature_pipeline.R.
  a. Confirm that the script completes without validation errors and writes Table S3, Figures 2A–2C, and Figure S1 to the results/directory.
  b. Verify that the generated table and figures reproduce the submitted outputs.

## Expected outcomes

This protocol enables the controlled and progressive accumulation of somatic mutations in A549-derived cell lines through iterative cycles of DNA damaging treatments. By combining defined cytotoxic stress (40%–60%) with recovery phases, the method supports long-term cell survival while promoting continuous mutagenesis, thereby modeling genome instability as a dynamic, time-dependent process.

During early treatment cycles (approximately 5–10 cycles), cells typically maintain relatively stable growth, with modest reductions in proliferation (∼10%–30%). At this stage, mutation burden remains limited, and phenotypic changes are subtle, although initial heterogeneity may begin to emerge. Following intermediate cycling (15–30 cycles), mutation accumulation becomes more pronounced. Cells frequently display increased variability in morphology and growth kinetics, and population doubling time may increase by ∼20%–50%, reflecting cumulative DNA damage and selection for damage-tolerant subpopulations. After extended treatment (50–70 cycles), surviving populations exhibit a markedly elevated mutational burden.

Among the 42 analyzed clones, WGS achieved mean depths of 20.31×-36.50× and mapping rates of 99.71%–99.88%, providing adequate data quality for the downstream analysis used here. One additional library failed sequencing quality control and was excluded before joint mutation calling. Complete per-clone sequencing quality metrics, including total read counts and duplicate rates, are provided in Table S2.

The extent of mutation accumulation depends strongly on the DNA damaging agent applied. After 70 iterative treatment cycles, untreated control clones contained a median of 3,137 filtered somatic SNVs (range: 971–5,948), representing the background mutation level acquired during long-term culture. In contrast, treated clones accumulated substantially higher numbers of mutations. Simulated solar radiation (SSR) generated a median of 16,687 SNVs per clone (range: 4,470–29,122), while cisplatin treatment resulted in a median of 4,986 SNVs (range: 1,160–15,891). Metabolically activated benzo[a]pyrene produced a markedly higher mutation burden, with a median of 65,089 SNVs per clone (range: 59,481–96,907), whereas repeated exposure to ENU generated the highest mutation burden, reaching a median of 266,616 SNVs per clone (range: 131,482–479,323). Overall, the protocol generated mutation burdens spanning approximately 10^3^ to nearly 5 × 10^5^ acquired SNVs per clonal genome, demonstrating that the extent of mutagenesis is highly dependent on the mutagenic mechanism.

The high mutation burden achieved through iterative treatment provides sufficient numbers of somatic variants for reliable mutational signature analysis. Comparison of SBS96 mutational spectra with experimentally derived SIGNAL reference signatures demonstrated that each treatment generated the expected mutational profile. UV-treated clones showed high similarity to the simulated solar radiation reference signature (median cosine similarity 0.92), cisplatin-treated clones matched the cisplatin reference (median 0.92), benzo[a]pyrene-treated clones displayed near-perfect agreement with the BPDE reference signature following metabolic activation by the S9 system (median 0.99), and ENU-treated clones closely matched the ENU reference signature (median 0.93). Untreated control clones showed a median cosine similarity of 0.77 to the prespecified Control reference, consistent with a heterogeneous endogenous and culture associated background spectrum.

These results demonstrate that the iterative treatment strategy preserves treatment-associated mutational profiles despite prolonged exposure while substantially increasing the number of mutations available for analysis. Consequently, the protocol provides sufficient mutation counts for high-resolution SBS96 profile construction and comparison with treatment-associated reference signatures.

***Note:*** Sequencing depth, mutation burden, and mutational signature similarity may vary depending on the cell type, DNA damaging agent, treatment intensity, sequencing platform, and bioinformatic analysis pipeline. However, maintenance of high sequencing quality together with reproducible treatment specific mutational signatures indicates successful implementation of the protocol.

Overall, this protocol reproducibly generates highly mutated, yet viable, cell populations that capture diverse mutational processes. These models are well suited for high-resolution mutational signature analysis, functional genomics, and studies of genome instability and its downstream consequences.

## Limitations

This protocol relies on iterative exposure to DNA damaging agents combined with recovery phases, which inherently selects for cells capable of tolerating repeated genotoxic stress. As a result, the final cell populations are enriched for damage resistant clones. Consequently, the observed mutational landscape reflects a combination of mutagen-induced DNA damage and selection pressures, and may not fully represent the unbiased spectrum of mutations generated by each agent.

Extended culture over prolonged periods (approximately 30–50 weeks) introduces additional biological variability. Genetic drift, epigenetic remodeling, and adaptation to in vitro growth conditions can influence cellular behavior and mutation profiles independently of treatment. These effects are particularly pronounced during later stages of the protocol (≥50 cycles), where population bottlenecks and clonal selection may reduce diversity and skew mutational patterns.

A critical limitation of this protocol is the requirement to maintain cytotoxicity within a relatively narrow range (40%–60%) throughout the iterative treatment cycles. Deviations from this range can substantially affect experimental outcomes: insufficient cytotoxicity may reduce mutation accumulation, whereas excessive cytotoxicity can lead to progressive cell loss and failure to sustain long-term treatment. The protocol has been optimized and experimentally validated in A549 human lung adenocarcinoma cells. Although the overall strategy is expected to be applicable to other mammalian cell lines, treatment conditions - including DNA damaging agent concentration, exposure duration, recovery time, and cell density - should be empirically optimized for each model, as cellular sensitivity to genotoxic stress varies considerably between cell types.

For compounds requiring metabolic activation, the use of the S9 system introduces an additional source of variability. Differences in enzymatic activity between S9 batches, as well as sensitivity to handling and storage conditions, can affect the reproducibility and efficiency of mutagenesis. This variability may influence downstream mutational signatures and should be carefully controlled.

Finally, the duration and labor-intensive nature of the protocol limit scalability. Maintaining consistent experimental conditions over many months is challenging and may introduce batch effects and operator dependent variability. These factors should be considered when designing experiments and interpreting results, particularly in comparative studies.

In addition, IsoMut cannot recover mutations shared between clones. The method identifies variants that are clone-specific relative to the other members of an isogenic panel. Any treatment induced mutation that arose before subcloning, and is therefore present in two or more final clones, is suppressed as background. Mutations present in the common ancestor of all clones are invisible to this workflow by construction. The reported burdens are consequently a lower bound on total treatment induced mutagenesis.

Reference catalogue choice materially affects attribution. COSMIC signatures are extracted from tumour genomes, in which multiple mutational processes operate simultaneously; some references are individually diffuse. ENU illustrates this: its treatment level spectrum matches COSMIC SBS5 at cosine 0.78, whereas it matches the SIGNAL experimental ENU signature at 0.93. Benchmark experimental exposures against experimentally derived signatures and treat COSMIC attribution as secondary context.

Cosine similarity can give moderately high values to diffuse references. Attribution should therefore emphasize prespecified experimental exposure signatures and the pattern across the full reference catalogue rather than a universal cosine cutoff.

Within-group heterogeneity can occur. Individual clones within a treatment group may diverge from the group signature. Where such heterogeneity appears, sequencing additional clones per condition helps establish whether the divergent clones represent a reproducible subpopulation.

This workflow was designed to evaluate agreement with prespecified experimental reference signatures, de novo signature extraction was not attempted.

Indels are not analysed. IsoMut reports indels, but only the SNV table is used here. Indel (ID-class) signatures are informative for some agents and were not evaluated.

Attribution quality is bounded by depth and mutation burden. This workflow was demonstrated at the observed depth and burden ranges; performance at substantially lower depth, very low burden, or after single dose exposure was not evaluated.

Untreated controls are not blank. NT clones carry a real, non-flat background spectrum compatible with endogenous or culture associated processes. Weakly mutagenic conditions must be interpreted against this background rather than against a featureless control.

Upstream steps were provider dependent. Alignment and duplicate marking were performed by the sequencing provider. Although any equivalent workflow is acceptable, differences in aligner version or duplicate marking behaviour propagate to the call set, and this sensitivity was not systematically assessed.

## Troubleshooting

### Problem 1: Excessive cell death following DNA-damaging treatment (related to steps 9–13)

High levels of cytotoxicity may result in near-complete loss of the cell population, particularly during early cycles when cells have not yet adapted to repeated genotoxic stress.

### Potential solution

• Perform preliminary dose-response experiments to accurately determine concentrations that induce 40%–60% cytotoxicity.
• Reduce treatment concentration or shorten exposure time if excessive cell death is observed.
• Ensure cells are in optimal condition prior to treatment (log-phase growth, ∼70% confluency).
• Avoid treating over-confluent or stressed cultures, as these are more sensitive to DNA damage.
• Confirm even distribution of treatment medium across wells to prevent localized overexposure.

### Problem 2: Poor recovery or lack of proliferation between treatment cycles (related to steps 14–16)

Cells may fail to recover after treatment, resulting in slow growth or progressive loss of the population across cycles.

### Potential solution

• Extend the recovery period beyond 2–4 days until cells demonstrate stable proliferation.
• Replace medium daily to remove residual toxic compounds.
• Reduce treatment intensity in subsequent cycles if recovery remains impaired.
• Ensure optimal culture conditions (temperature, medium quality, serum quality).
• Consider splitting cells at lower density to reduce stress during recovery.

### Problem 3: Inconsistent mutation accumulation between replicates or treatment conditions (related to steps 17–20)

Variation in mutation burden may arise from both technical factors (e.g., inconsistencies in treatment conditions across cycles) and biological factors, including differences in the mutagenic potency of individual DNA damaging agents, stochastic mutation acquisition, and clonal selection during long-term culture.

### Potential solution

• Perform at least three independent biological replicates for each treatment condition.
• Standardize cell seeding density, treatment timing, recovery duration, and culture conditions across all treatment cycles.
• Use the same batches of reagents whenever possible and record experimental metadata throughout the experiment.
• Compare mutation burden only between experiments performed under identical conditions and with equivalent numbers of treatment cycles.
• To distinguish technical variability from biological variability, evaluate the reproducibility of mutation burden and mutational signature profiles across independent replicates. Consistent mutational signatures with variable mutation counts are more likely to reflect biological differences between DNA damaging agents rather than experimental inconsistency.
• If substantial differences are observed between biological replicates treated under identical conditions, reassess cytotoxicity, treatment timing, and cell viability throughout the iterative treatment protocol.

### Problem 4: Reduced or variable activity of S9 metabolic activation system (related to step 8)

Loss of S9 enzymatic activity can lead to inefficient activation of pro-mutagens such as benz[a]pyrene.

### Potential solution

• Prepare S9 mix fresh immediately before each use.
• Store S9 fraction in small aliquots to prevent repeated freeze-thaw cycles.
• Keep S9 components on ice during preparation and handling.
• Verify S9 activity using a known positive control if inconsistent results are observed.
• Avoid prolonged incubation of S9 mix before application to cells.

### Problem 5: Failure of clonal expansion after single-cell isolation (related to steps 21–24)

Single cells may not survive or expand efficiently following extensive treatment cycles.

### Potential solution

• Optimize seeding density to ensure true single-cell conditions while maintaining viability.
• Use conditioned medium or increase serum concentration to support initial growth.
• Select cells during logarithmic growth phase prior to cloning.
• Avoid cloning immediately after treatment; allow at least one full recovery cycle.
• Minimize handling stress during isolation and early expansion.

### Problem 6: Progressive loss of cell population during long-term cycling (related to step 17)

Extended exposure to DNA damaging agents may lead to cumulative stress and collapse of the culture.

### Potential solution

• Cryopreserve intermediate populations at regular intervals (e.g., every 5–10 cycles).
• Monitor cell morphology and growth rates to detect early signs of decline.
• Adjust treatment intensity or increase recovery time if viability decreases over time.
• Maintain backup cultures to prevent complete experimental loss.
• Avoid overly aggressive passaging or handling during later cycles.

### Problem 7: Low number of detected mutations after sequencing

Mutation burden may be lower than expected, resulting in noisy SBS96 profiles and unstable comparison with reference signatures.

### Potential solution

• Verify that cytotoxicity levels were within the target range (40%–60%) during treatment cycles.
• Increase the total number of treatment cycles if mutation accumulation is insufficient.
• Confirm sequencing depth (∼30× coverage) and variant calling sensitivity.
• Review filtering criteria to ensure true variants are not being excluded.
• Validate effectiveness of DNA damaging agents using control assays if needed.

### Problem 8: Contamination or mixed clones during clonal isolation (related to steps 21–24)

Non-monoclonal populations can compromise downstream sequencing and interpretation.

### Potential solution

• Carefully select well-isolated colonies during clonal expansion.
• Avoid picking colonies that are in close proximity or overlapping.
• Confirm monoclonality by visual inspection and, if necessary, genotyping.
• Reduce initial seeding density to improve colony separation.
• Repeat cloning if mixed populations are suspected.

### Problem 9

Variants disappear silently between mutation calling and profile construction (related to steps 32, 35). Downstream steps run without error, but far fewer variants reach the SBS96 matrix than the IsoMut output contains.

### Potential solution

A common cause is a chromosome naming mismatch between the BAM, IsoMut reference, call set, blacklist, and BSgenome object. Inspect the BAM header:

samtools view -H bams/sample.final.bam | grep 'ˆ@SQ' | head

Inspect the call set:

cut -f2 isomut_output/all_SNVs.isomut | sort -u | head

Inspect the R genome object:

head(seqlevels(BSgenome.Hsapiens.UCSC.hg38))

Confirm that all three use compatible chromosome names. Do not mix Ensembl-style names (1…Y) with UCSC-style names (chr1…chrY); use one compatible reference packaging throughout.

### Problem 10

A clone yields an implausibly low mutation count, or IsoMut calls large numbers of mutations in untreated controls (related to steps 29, 31).

### Potential solution

Check mapping rate and mean depth for the clone before anything else; a failed library can carry a normal number of raw reads while yielding almost no aligned genome (see step 29 CRITICAL). Confirm all clones were called in a single IsoMut execution; separate runs remove the cross-clone comparisons that suppress background. Confirm untreated controls were sequenced in the same batch at comparable depth; depth imbalance degrades the min_other_ref_freq criterion. Verify blacklist filtering was applied (step 33). Inspect the cleanliness column: a preponderance of low-cleanliness calls indicates the panel is not behaving as an isogenic set.

### Problem 11

A treatment spectrum matches no reference convincingly, or several unrelated signatures score similarly (related to step 38).

### Potential solution

Check burden first; low counts give noisy spectra and unstable attribution. Confirm that comparison is made against the SIGNAL experimental catalogue rather than COSMIC alone. Consider whether the expected reference is the applied parent compound or a reactive metabolite: benzo[a]pyrene treated with S9 can match BPDE, its DNA reactive metabolite, more strongly than the parent-compound reference. Also check for dose-specific entries because SIGNAL contains several concentrations of some agents.

### Problem 12

SIGNAL agent names look implausible (related to step 37). Names such as 1.8 DNP or 5 Methylchrysene appear, and searching the SIGNAL website for “AAII” returns nothing.

### Potential solution

get_known_signatures() returns names in R make.names() form, where punctuation is replaced by periods. Do not attempt to reverse the transformation with a regular expression because it is not invertible. Keep native names for computation and use the supplied validated display labels only where needed. For example, 1.8 DNP corresponds to 1,8-dinitropyrene, and AAI/AAII denote aristolochic acid I/II. Consult Kucab et al.^2^ for names and doses.

### Problem 13

Clones within a treatment group show divergent SBS96 profiles (related to steps 35, 38, and 39).

### Potential solution

Check for a burden outlier within the group; a clone with an order-of-magnitude lower burden may have a noisy spectrum. Confirm that clones are single-cell-derived and have completed the full treatment schedule. Fewer treatment cycles may reduce mutation burden or alter reproducibility, but they do not predict a specific position between the control and treatment profiles. Genuine heterogeneity should be investigated rather than suppressed.

## Resource availability

### Lead contact

Further information should be directed to and will be fulfilled by the lead contact, Szilvia Juhász (juhasz.szilvia@brc.hu).

### Technical contact

Technical questions should be directed to Szilvia Juhász (juhasz.szilvia@brc.hu).

### Materials availability

This study did not generate new unique reagents.

### Data and code availability

The data analyzed in this protocol derive from Juhász et al.^1^ Sequencing reads for the publicly deposited subset are available in the ENA under accession PRJEB102539. Table S1 lists, for each clone, the protocol name, original clone ID, ENA BioSample accession, and availability status. Sequencing data for additional clones are available from the lead contact upon request.

The joint IsoMut SNV call set used as input for the reported downstream R analysis is available as input/all_SNVs.isomut.gz in the project GitHub repository (https://github.com/imgergely/WGS-signatures). The repository also contains the SAMtools quality control script (run_samtools_qc.sh); IsoMut configuration (isomut_config.py); downstream R pipeline (wgs_signature_pipeline.R); and clone key, sequencing quality control table, mutation count table, and associated figures. Version 1.0.0 of the repository has been archived as the version of record on Zenodo: https://doi.org/10.5281/zenodo.22001044.

## Acknowledgments

This work was supported by 10.13039/501100011019The National Research, Development and Innovation Office, Hungary (NKFIH) KIM NKFIA TKP-2021-EGA-05 (S.J.) and The National Research, Development and Innovation Office, Hungary (NKFIH) KIM NKFIA 2022-2.1.1-NL-2022-00005 (S.J.). H2020-WIDESPREA-01-2016-2017-TeamingPhase2, GA:739593-HCEMM, EU’s Horizon 2020 research and innovation program under grant agreement No. 739593 (S.J.).

## Author contributions

S.J. conceived and designed the protocol, developed and optimized the cyclic DNA damaging treatment strategy, and wrote this manuscript. G.I. performed the mutational signature analysis.

## Declaration of interests

The authors declare no competing interests.
