# Supplementary material for: Protocol for cyclic DNA-damaging treatments to generate high mutational burden in the human A549 cell line
Source: STAR Protoc. 2026 Sep 18;7(4):104842. doi: 10.1016/j.xpro.2026.104842 (PMC13602311; doi:10.1016/j.xpro.2026.104842)
Supplement: Supplementary file 1 [file mmc1.pdf]

Zur optimalen Anzeige dieses PDF-Portfolios sollte es in Acrobat oder Adobe Reader ab Version X geöffnet werden.

Adobe Reader jetzt herunterladen
